# Long-Term Efficacy of Prism Adaptation on Spatial Neglect: Preliminary Results on Different Spatial Components

**DOI:** 10.1100/2012/618528

**Published:** 2012-05-02

**Authors:** Maria Luisa Rusconi, Laura Carelli

**Affiliations:** Department of Human Sciences, University of Bergamo, 24129 Bergamo, Italy

## Abstract

This study describes the long-term effectiveness on spatial neglect recovery of a 2-week treatment based on prism adaptation (PA). Seven right-brain-damaged patients affected by chronic neglect were evaluated before, after two weeks of the PA treatment and at a follow-up (variable between 8 and 30 months after the end of PA). Neglect evaluation was performed by means of BIT (conventional and behavioral), Fluff Test, and Comb and Razor Test. The results highlight an improvement, after the PA training, in both tasks performed using the hand trained in PA treatment and in behavioral tasks not requiring a manual motor response. Such effects extend, even if not significantly, to all BIT subtests. These results support previous findings, showing that PA improves neglect also on imagery tasks with no manual component, and provide further evidence for long-lasting efficacy of PA training. Dissociations have been found with regard to PA efficacy on peripersonal, personal, and representational neglect, visuospatial agraphia and neglect dyslexia. In particular, we found no significant differences between the pre-training and post-training PA session in personal neglect measures, and a poor recovery of neglect dyslexia after PA treatment. The recruitment of a larger sample could help to confirm the effectiveness of the prismatic lenses with regard to the different clinical manifestations of spatial neglect.

## 1. Introduction

Unilateral spatial neglect (USN) is a syndrome more frequently observed in patients with right brain damage who often do not report or respond to stimuli presented in the contralesional hemispace despite the absence of elementary sensory or motor deficits [1–3]. It is characterized by different clinical manifestations involving different portions of space, that is, personal [4, 5], peripersonal [6], and extrapersonal [7]. USN is not limited to the visual domain and may also occur for auditive, olfactory, tactile, and somatosensory stimuli, and it can even affect the contralesional side of mental representations [8].

Neglect symptoms are observed in at least 50% of all acute patients with right brain damage and though most patients recover spontaneously in the acute poststroke phase [9], chronic neglect is severely debilitating and difficult to rehabilitate [10]. Therefore, an effective treatment for chronic neglect is much needed.

Several studies have investigated different rehabilitation approaches for spatial neglect over the last decades. Some of these procedures have focused on top-down based mechanisms (e.g., [11–13]), while others have been based on bottom-up strategies such as vestibular, optokinetic, transcutaneous and proprioceptive stimulation and transcranial magnetic stimulation (e.g., [14–19]). These lateralized or directionally specific physiological stimulations may temporarily ameliorate a great number of USN manifestations.

Among the rehabilitation studies using behavioral training to enhance the exploration of the left-hand side of space or physiological stimulation, the prism adaptation (PA) [20] has shown that the improvement of the USN syndrome can be more long-lasting. The authors found that a brief period of simple visuomotor adaptation to a lateral shift of the visual field (prism adaptation) ameliorated visual neglect symptoms for two hours. Subsequently, Pisella et al. [21] reported that the symptom reduction could persist for several days, and later studies have shown an effect of PA lasting for five to twelve weeks after training [22, 23]. More recent studies [24, 25] have reported a more lasting beneficial effect on USN, that is, up to six months after the prism application.

In PA procedure, patients are trained on a pointing task while wearing prismatic goggles inducing a 10° rightward deviation of the visual field. This change in sensory input determines a modification of perceptual and motor representations of external space inducing visuomotor responses that are misdirected to the right. When the prisms are removed, this adaptation process causes an overcompensation by pointing too far to the left (the after effect).

Improvements have been observed not only in paper-and-pencil neglect tasks (line cancellation, line bisection, and drawing by copying or by memory) but also in visuoverbal tasks (object description, object naming, and word and nonword reading) [22, 26], naming towns from a mental map [26], and awareness of tactile stimulations [27].

These findings suggest that the long-lasting effect of prism procedure may be conceived as improving active processes involved in brain plasticity related to multisensory integration and high-level space representation.

All these interesting studies have observed a beneficial effect on USN for a period variable between two weeks and six months after the PA. Recently, Shiraishi et al. [28] reported a possible positive long-lasting prism effect in five chronic neglect patients, at 2–3.5 years after the end of prism application. Their results showed that the beneficial effect was not limited to cancellation or line bisection tasks, but also involved ecological aspects measured by ADL (basic activities of daily living) performance. However, the authors did not use other instruments useful to investigate the different neglect manifestations, such as personal neglect, representational neglect, neglect dyslexia, and behavioral aspects (by means of BIT behavioral subtest).

In our study, starting from the observation of previous works, we describe seven chronic right-brain-damaged patients affected by different clinical neglect manifestations. The main aim was to evaluate the efficacy of PA training in a long-term follow-up (variable from 8 to 30 months after the prism application) about the different clinical neglect manifestations, that is, personal/peripersonal/representational neglect, neglect dyslexia, and agraphia by means of a standardized battery (BIT, conventional and behavioral) and specific personal neglect test (Fluff Test [29] and Comb and Razor Test [30]).

## 2. Method

### 2.1. Subjects

We recruited seven patients with unilateral spatial neglect consequent to right cerebral hemisphere vascular lesions. The onset of illness ranged from two and six months. The sample was composed by three males and four females, with a mean age of 45.14 years, DS 16.36 years (range 17–65), all right-handed. The mean level of education was 9.28 years, DS 3.4 years (range 8–17) (see Table 1). Patients have not received any other cognitive treatment during the assessment period.

### 2.2. Neuropsychological Evaluation

Patients were submitted to the behavioral inattention test battery [31] which includes conventional subtests (*line crossing, letter cancellation*, *stars cancellation*, *figures and shape copying*, *line bisection*, and* drawing from memory*) and behavioral subtests (*picture scanning*, *telephone dialling*, *menu reading*, *article reading*, *telling and setting the time*, *coins and cards sorting*, *address and sentence copying*, and* map navigation*). The cut-off scores of the conventional and behavioral subtests are 129 (0–146, maximum score 146) and 67 (0–81, maximum score 81), respectively. Patients were classified as having neglect if they obtained scores below the cut-off score in at least one subtest. The presence of different neglect deficits was established on the basis of the cut-off obtained from the individual subtests: neglect for peripersonal space (by means of conventional subtests); representational neglect (*representational drawing* subtest); neglect dyslexia (*article reading* and *menu reading subtest*); visual-spatial agraphia (*address and a sentence copy subtest*).

The following tests were also employed in order to investigate the presence of personal neglect: *Fluff Test* and *Comb and Razor Test*. In the Fluff test, patients were blindfolded and seated, whilst the experimenter attached six pieces of adhesive paper to their clothing on the left part of their body. Once the blindfold had been removed, patients were required to remove all pieces of paper within two minutes. The number of omitted pieces was recorded. A score below 13 suggests the presence of neglect for the left personal space. The Comb and Razor Test screens for unilateral spatial neglect in patients' personal space by assessing their performance in functional activities, such as using a comb or applying make-up. In such test, patients were asked to demonstrate the use of common objects (comb and razor or powder compact) for 30 seconds. Each object was placed in correspondence of the patient's mid-line. The number of strokes with the razor, comb or powder compact that are performed on the left or right side or ambiguously were recorded to calculate a mean percentage score for the three categories. A score below 0.35 indicates the presence of left personal neglect.

### 2.3. Prism Adaptation Training

The prism adaptation training followed the procedure laid out by Serino et al. [23]. Patients were seated at a table on which there was a wooden box, open on the side facing the patient and on the opposite side, facing the experimenter. The task was to point to a visual target with the right index finger. The visual targets were presented randomly at one of three positions, either directly in front of the patient or 21° to the left or to the right. Patients performed half of the trials with visible pointing and half with invisible pointing (pointing movement was executed below the top face of the wooden box), and the experimenter noted deviations in the patients' pointing accuracy.

Each training session consisted of three different conditions. In the *pre-exposure* condition, the pointing was performed without goggles. Pointing was first done with no visual feedback of performance to obtain a measure of the baseline level of pointing accuracy and then with visual feedback, and 60 targets were randomly presented at one of three possible positions (20 targets in the centre, 20 on the right, 20 on the left).

In the *exposure* condition, patients wore prismatic goggles creating a rightward optical shift of 10° and performed 90 pointing movements (30 targets in the centre, 30 on the right, and 30 on the left) while receiving visual feedback on the landing position of the finger.

 In the *post-exposure* condition, after removal of the prism, patients were required to point, below the top face of the box, towards 30 targets (10 in the centre, 10 on the right, 10 on the left).

### 2.4. Procedure

The study consisted of four steps.

- Pre-treatment neuropsychological evaluation: all patients were first evaluated by means of the described battery in a chronic phase of the disease (at least 2 months of length of illness, that is, time-interval between stroke and neuropsychological evaluation), in order to verify the presence of neglect.
- Prism adaptation (PA) training: patients were submitted to a training with prismatic lenses; the training, lasting two weeks (from Monday to Friday), was carried out twice a day, and each session lasted about 20 minutes.
- Post-treatment neuropsychological evaluation: subjects were retested through BIT, Comb and Razor Test, and Fluff Test after 14 days of completion of the rehabilitation treatment with prismatic lenses, with the aim to evaluate the possible improvements of neglect in the different sectors of the space.
- Follow-up: after a period ranging from eight to thirty months patients were retested with the so-defined battery, in order to evaluate the long-lasting effect of the PA treatment.

### 2.5. Statistical Analysis

Data were collected with regard to patient's scores on BIT (conventional and behavioral subtests), Fluff Test, and Comb and Razor Test.

A nonparametric statistical test for repeated measurements (the Wilcoxon signed-rank test) was employed, in order to compare scores obtained at the different successive neuropsychological evaluations.

In particular, a comparison was made between pre-training and post-training, post-training and follow-up and pre-training and follow-up scores obtained in the BIT (conventional and behavioral subtests), in Fluff test and Comb and Razor test. Comparison between pre-training and post-training neuropsychological, evaluation was performed to detect the presence of treatment-related improvements and highlight the tasks where these improvements had occurred. A further comparison between post-training evaluation and follow-up was performed, in order to evaluate whether the improvements due to PA training had remained stable over time. Finally, the comparison between the pre-training and follow-up was designed to highlight any minor increase or decrease in scores between the different evaluations. 

## 3. Results

### 3.1. Descriptive Statistics

Table 2 shows means (M) and standard deviation (SD) in the several subtests with regard to the three successive evaluations (pre-, post-treatment, and follow-up).

### 3.2. Clinical Neglect Evaluation with Regard to Pre-Treatment, Post-Treatment, and Follow-up Results

In Table 3, the clinical manifestations of neglect presented by patients at the time of initial assessment and in the two subsequent evaluations (post-training and follow-up) are depicted. First, we can observe that, 14 days after treatment, only 1/7 patients no longer showed deficitary scores in any subtest, and 4/7 patients showed a complete recovery in the follow-up evaluation. With regard to *personal neglect*, only 3/7 patients presented deficitary scores in the Fluff Test or Comb and Razor test in the initial assessment; one of them (*n*.5) obtained a long-lasting recovery after PA training, another one (*n*.4) showed a complete recovery in the follow-up, and only one patient (*n*.6) still presented with the disorder at the follow-up evaluation.

 The only patient (*n*.6) who manifested representational neglect in the initial assessment showed a total recovery at 14 days after treatment and maintained this improvement over time, as showed by follow-up evaluation. With regard to the presence of visual-spatial agraphia, 5/7 patients had this disorder at the initial evaluation, 4/5 of them showed normal performances in the related BIT subtests in the post-training assessment, and recovery was complete for all patients at the follow-up. Instead, with regard to neglect dyslexia, the deficit was present in 7/7 patients initially, with only 2/6 patients showing a complete, and long-lasting, recovery in the post-training assessment, while three patients were still impaired in the follow-up.

### 3.3. Comparison between Pre- and Post-Training Assessment

**Figure 1:**
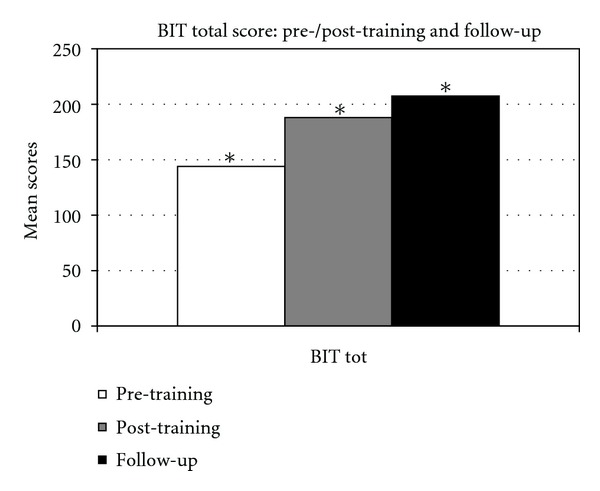
BIT total score in the pre-/post-training and in the follow-up evaluation. *Significant value.

Regarding the statistical comparison carried out on results obtained in the pre- and post-training evaluation, a significant improvement has been observed in BIT total scores (conventional and behavioral subtests), *z* = 2.36, *P* < .05 (see Figure 1).

**Figure 2:**
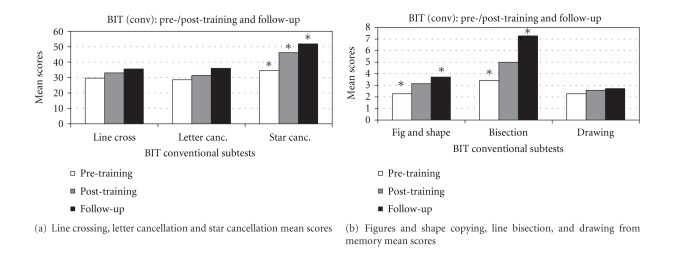
BIT conventional subtests scores in the pre-/post-training and in the follow-up evaluation. *Significant value.

**Figure 3:**
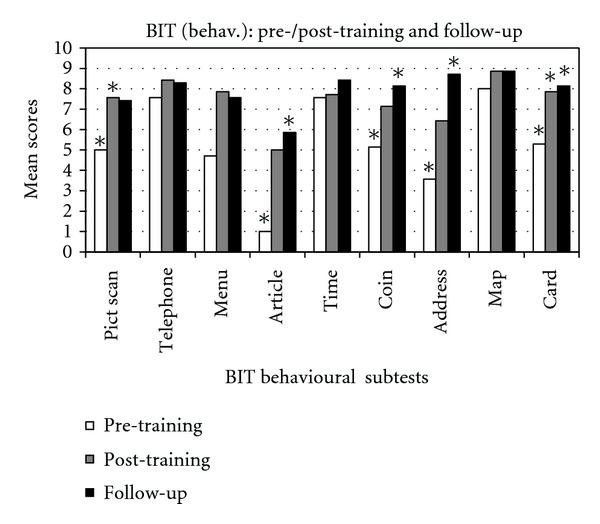
BIT behavioral subtests scores in the pre-/post-training and in the follow-up evaluation. *Significant value.

**Figure 4:**
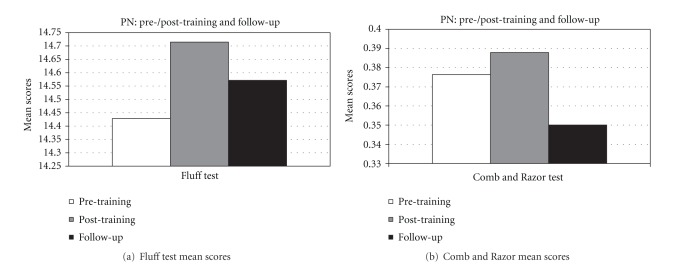
Personal neglect tests in the pre-/post-training and in the follow-up evaluation.

Moreover, we found a statistically significant improvement in the following subtests: *picture scanning* (*z* = 2.02, *P* < .05) and *cards sorting* (*z* = 2.02, *P* < .05). By considering the mean values obtained at the subtests of BIT, at the Fluff test and Comb and Razor Test, an improvement, although not significant, is present for all tasks when comparing pre- and post-treatment assessment (Figures 2, 3, and 4).

### 3.4. Comparison between Post-Training and Follow-up

By comparing Post-training and follow-up evaluation scores, a significant improvement has been shown with regard to BIT total scores (*z* = 2.36, *P* < .05) and in the *stars cancellation *subtest (*z* = 2.20, *P* < .05). Moreover, an increase in the mean values, although not significant, has been observed in the following subtests: *line crossing*, *letter cancellation*, *stars cancellation*, *figures and shape copying*, *line bisection*, *drawing from memory*, *article reading*, *telling and setting the time*, *coins sorting*, *address and sentence copying*, *and cards sorting *(Figures 2 and 3). No changes are noticed in the subtest *map navigation*. On the other side, a decrease of average scores has been observed in some BIT subtests, that is, *picture scanning*, *menu reading*, *and telephone dialling *(Figures 2 and 3) and in test for personal neglect (Figure 4).

### 3.5. Comparison between Pre-Training and Follow-up

The comparison between pre-training and follow-up evaluation highlights a statistically significant improvement in the following subtests: BIT total score, *z* = 2.36, *P* < .05; *star cancellation*, *z* = 2.20, *P* < .05; *figures and shape copying*, *z* = 2.36, *P* < .05; *line bisection, z* = 2.36, *P* < .05; *article reading*, *z* = 2.20, *P* < .05*; coins sorting, z* = 2.02, *P* < .05; *address and sentence copy*, *z* = 2.02, *P* < .05; *cards sorting, z* = 2.02, *P* < .05.

Furthermore, by considering the average scores, an increase, although not statistically significant, has been observed in the follow-up evaluation in all other subtests of the BIT (*line crossing*, *letter cancellation*, *telephone dialling*, *menu reading*, *telling and setting the time*, *and map navigation*) and in the Fluff test; a slight decrease of mean score has been observed in Comb and Razor test (Figures 2, 3, and 4).

## 4. Discussion

 Our results highlight an improvement, after the PA training, in both visuo-spatial/motor tasks, similar to those addressed during the treatment, and behavioral tasks without a manual motor response (such as *picture scanning*). In fact, an improvement has been observed after PA training in most subtests, and resulted to be long-lasting, according to the follow-up evaluation. Moreover, an increase has been found in mean scores at the different subtests across sessions.

These results support previous findings, showing that prism adaptation improves neglect on imagery tasks with no manual component [32]. They also support the view that prism adaptation induces changes also at higher cognitive levels of spatial representation, and not only in visuomotor coordination [20, 22, 23, 32, 33]. Rossetti et al. [20], in order to explain the efficacy of PA, have suggested that it works as a lateralized alarm signal, consequent to a visual proprioceptive discrepancy between the expected hand position and that observed, which could activate a compensatory cerebral mechanism. Other authors have proposed the presence of ocular motor changes, that could influence spatial representations. Such hypothesis has been then confirmed by successive studies, which have highlighted an increase in ocular movements toward the neglected side after a PA training [23, 28]. With regard to the present study, even though we have not found significant differences between scores in *representational drawing* subtest across sessions, the only patient with representational neglect showed a complete and long-lasting recovery after PA training. This could suggest a reorganization in high-order visuospatial representations, even if, unfortunately, an ocular movements quantitative measurement has not been performed in our study, since the eye-tracker technology was not available.

With regard to personal neglect, no significant differences between the pre-training and post-training PA session have been observed in the *Fluff Test* and *Comb and Razor Test* scores. However, only 3/7 patients presented impaired performances in those tests in the initial assessment; one of them obtained a long-lasting recovery after PA training, another one showed a complete recovery in the follow-up and only one still presented with the disorder at the follow-up evaluation. Due to the paucity of patients showing personal neglect, we cannot draw reliable conclusions with regard to the efficacy of PA training for such disease.

Finally, 5/6 patients with visual spatial agraphia at the pre-training evaluation no longer showed such disturbance in the post-treatment session, and all of them had completely recovered at the follow-up. Differently, only 2/6 patients with neglect dyslexia had recovered after PA training with long-lasting improvements. Some authors had shed light on anatomical correlates of neglect dyslexia; in particular, in a recent review by Vallar et al. [34], the authors highlighted that lesions involving the temporo-parietal-occipital regions are present in a large proportion of patients with ND, while lesions to the frontal lobe are much infrequent. Moreover, ND patients, compared to USN patients without ND, have additional lesions in the lingual and fusiform gyri. These findings suggest that ND is a specific component of the USN syndrome, brought about by posterior damage. Our results are not fully in accord with these findings; in fact, all patients in our group showed ND, five of them had lesion sites which involved the frontal regions, and only two of them had occipital lesions. However, no concluding considerations can be carried out from our research, since a thorough investigation of brain damages has not been conducted. Moreover, while several studies have found significant improvements in neglect dyslexia after PA training [22, 23], we did not observe such an effect in our work. However, differently from those studies, we did not employ for ND assessment specific word reading tests, but two BIT subtests (*menu* and *article reading*). Similarly to our study, McIntosh et al. [33] employed a poem reading task in their research and found no significant effect of PA on neglect dyslexia. So, it could be that the use of a measure such as the reading of a text may overestimate the presence of reading errors. This could also explain why all patients in our sample showed the presence of neglect dyslexia at the pre-training evaluation.

Serino et al. [23], by investigating anatomical correlates of PA efficacy, found a correlation between a poor neglect recovery after PA treatment and frontal and occipital lobe lesions. Moreover, it has been demonstrated that the vast majority of patients presenting chronic neglect are affected by extended frontal lesions [35]. With regard to our study, 5/7 patients had lesions involving the frontal lobes, and two of them showed a good outcome after PA treatment at the *follow-up* evaluation. However, only one of them showed a total recovery of neglect just after the PA treatment. Even if these results should be carefully considered, since no exact calculation has been conducted about the proportion of damaged brain regions, it could be that, by extending the follow-up period, new evidences arise with regard to relationship between PA and neuroanatomical correlates.

Recent studies have confirmed the long-term efficacy of PA training as a rehabilitation treatment for unilateral spatial neglect [22–25, 36]. A recent work in this field of study has shown that amelioration of neglect consequent to PA training lasts up to two years after the end of the treatment in patients in the chronic stage of the disease [28].

The present work has provided further evidence for long-lasting efficacy of PA training in a sample of seven patients with chronic unilateral spatial neglect with a follow-up period ranging from eight to thirty months. Dissociations have been found with regard to PA efficacy on peripersonal/personal/representational neglect, visuospatial agraphia and neglect dyslexia. In particular, a poor recovery of neglect dyslexia has been observed after PA treatment, compared to results presented by available literature, even if methodological issues, like the use of different assessment measures, could partially explain such differences. The recruitment of a larger sample could help to confirm the effectiveness of the prismatic lenses with regard to personal and representational neglect, which were less represented in the sample recruited. Finally, it would be interesting to investigate in more depth the relationship between the lesion sites and PA efficacy.

Our study is not placebo-controlled, and it can be considered a main limitation. However, since our patients were in the chronic phase of illness and no other cognitive training were administered during the successive evaluations, we can reasonably argue that the observed changes have been consequent to the PA training administration. The recruitment of a group of patients with a variable duration of illness may reinforce such assumption. We cannot exclude, however, a possible effect of spontaneous fluctuations of neglect severity. Due to the small number of subjects included in the study, our results about long-lasting efficacy of prism adaptation may be quite limited, even if promising.

## Figures and Tables

**Table 1:** Demographical and clinical data of right brain damaged patients.

ID	Gender	Age	Educ. (years)	Lesion site	Etiology	Onset (months)
1	F	38	8	T, O, Ins., Int. Cap.	H	6
2	M	17	8	F, T, Ins., Lent.	I	3
3	F	35	8	F, T, P	I	2
4	M	48	8	T, P, O	H	2.5
5	F	57	8	F, T, Ins.	H	2
6	M	65	17	F, T	I	3
7	F	56	8	F	H	4

Etiology: I: ischemic; H: hemorrhagic.

Lesion site: F: frontal; T: temporal; P: parietal; O: occipital; Ins: insula; Int. cap.: internal capsule; Lent.: lenticular nucleus.

**Table 2:** Mean (M) and standard deviation (SD) values for pre-treatment, post-treatment, and follow-up evaluation.

Test	M pre	SD pre	M post	SD post	M follow-up	SD follow-up
Bit Tot	144.00	58.34	188.14	51.76	207.42	26.09
Line cross	29.57	8.84	33.00	7.07	35.57	0.53
Letter canc.	28.57	8.59	33.42	11.31	36.00	6.95
Star canc	34.57	19.23	46.14	12.74	51.85	3.23
Figure and shape	2.28	1.11	3.14	1.46	3.71	0.75
Bisection	3.42	2.22	5.00	3.46	7.28	2.05
Drawing	2.28	0.75	2.57	0.53	2.71	0.48
Picture scan	5.00	3.60	7.57	2.29	7.42	1.51
Telephone	7.57	1.39	8.42	0.97	8.28	1.25
Menu	4.71	4.34	7.85	3.02	7.57	2.99
Article	1.00	2.64	5.00	4.43	5.85	4.25
Time	7.57	2.14	7.71	1.60	8.42	1.13
Coins	5.14	3.76	7.14	3.28	8.14	2–26
Address	3.57	4.03	6.42	3.55	8.71	0.48
Map	8.00	2.64	8.85	0.37	8.85	0.37
Cards	5.28	3.54	7.85	3.02	8.14	2.26
Fluff test	14.42	1.13	14.71	0.48	14.57	1.13
Comb and Razor	0.37	0.13	0.38	0.15	0.35	0.14

**Table 3:** Clinical neglect evaluation at pre-training, post-training, and follow-up evaluation.

ID	Pre-training	Post-training	Follow-up (months)
1	PPN+ ND+	PPN+	N− (9)
2	PPN+ ND+ A+	N−	N− (11)
3	ND+ A+	ND+	ND+ (15)
4	PPN+ PN+ ND+ A+	PN+ ND +	N− (24)
5	PPN+ PN+ ND+	PPN+ ND+	ND+ (8)
6	PPN+ PN+ Repr. N+ ND+ A+	PPN+ PN+ ND+ A+	PPN+ PN+ ND+ (9)
7	PPN+ ND+ A+	ND+	N− (30)

PPN: peripersonal neglect; PN: personal neglect; Repr. N: representational neglect; ND: neglect dyslexia; A: visuospatial agraphia; N−: absence of neglect. + present; − absent.

## References

[1] Heilman KM, Heilman KE, Valenstein E, Hubley AM, Tremblay D (1993). Neglect and related disorders. *Clinical Neuropsychology*.

[2] Mesulam MM (1999). Spatial attention and neglect: parietal, frontal and cingulate contributions to the mental representation and attentional targeting of salient extrapersonal events. *Philosophical Transactions of the Royal Society B*.

[3] Bisiach E, Vallar G, Boller F, Grafman J (2000). Unilateral neglect in humans. *Handbook of Neuropsychology*.

[4] Bisiach E, Perani D, Vallar G, Berti A (1986). Unilateral neglect: personal and extra personal. *Neuropsychologia*.

[5] Guariglia C, Antonucci G (1992). Personal and extrapersonal space: a case of neglect dissociation. *Neuropsychologia*.

[6] Ladavas E, Farnè A, Spence C, Driver J (2004). Neuropsychological evidence for multimodal representations of space near specific body parts. *Crossmodal Space and Crossmodal Attention*.

[7] Halligan PW, Marshall JC (1991). Left neglect for near but not far space in man. *Nature*.

[8] Bisiach E, Luzzatti C (1978). Unilateral neglect of representational space. *Cortex*.

[9] Farnè A, Buxbaum LJ, Ferraro M (2004). Patterns of spontaneous recovery of neglect and associated disorders in acute right brain-damaged patients. *Journal of Neurology, Neurosurgery and Psychiatry*.

[10] Robertson IH (1999). Cognitive rehabilitation: attention and neglect. *Trends in Cognitive Sciences*.

[11] Antonucci G, Guariglia C, Judica A (1995). Effectiveness of neglect rehabilitation in a randomized group study. *Journal of Clinical and Experimental Neuropsychology*.

[12] Barrett AM, Buxbaum LJ, Coslett HB (2006). Cognitive rehabilitation interventions for neglect and related disorders: moving from bench to bedside in stroke patients. *Journal of Cognitive Neuroscience*.

[13] Pizzamiglio L, Antonucci G, Judica A, Montenero P, Razzano C, Zoccolotti P (1992). Cognitive rehabilitation of the hemineglect disorder in chronic patients with unilateral right brain damage. *Journal of Clinical and Experimental Neuropsychology*.

[14] Karnath HO (1994). Subjective body orientation in neglect and the interactive contribution of neck muscle proprioception and vestibular stimulation. *Brain*.

[15] Pierce SR, Buxbaum LJ (2002). Treatments of unilateral neglect: a review. *Archives of Physical Medicine and Rehabilitation*.

[16] Rossetti Y, Rode G, Vallar G (2002). Reducing spatial neglect by visual and other sensory manipulations. *Non Cognitive Routes to the Rehabilitation of a Cognitive Disorder*.

[17] Rubens AB (1985). Caloric stimulation and unilateral visual neglect. *Neurology*.

[18] Vallar G, Guariglia C, Rusconi ML, Thier P, Karnath H-O (1997). Modulation of the neglect syndrome by sensory stimulation. *Parietal Lobe Contributions to Orientation in 3D Space*.

[19] Koch G, Bonnì S, Giacobbe V (2011). Theta-burst stimulation of the left hemisphere accelerates recovery of hemispatial neglect. *Neurology*.

[20] Rossetti Y, Rode G, Pisella L (1998). Prism adaptation to a rightward optical deviation rehabilitates left hemispatial neglect. *Nature*.

[21] Pisella L, Rode G, Famè A, Boisson D, Rossetti Y (2002). Dissociated long lasting improvements of straight ahead pointing and line bisection tasks in two hemineglect patients. *Neuropsychologia*.

[22] Frassinetti F, Angeli V, Meneghello F, Avanzi S, Làdavas E (2002). Long-lasting amelioration of visuospatial neglect by prism adaptation. *Brain*.

[23] Serino A, Angeli V, Frassinetti F, Làdavas E (2006). Mechanisms underlying neglect recovery after prism adaptation. *Neuropsychologia*.

[24] Luauté J, Schwartz S, Rossetti Y (2009). Dynamic changes in brain activity during prism adaptation. *Journal of Neuroscience*.

[25] Serino A, Bonifazi S, Pierfederici L, Làdavas E (2007). Neglect treatment by prism adaptation: what recovers and for how long. *Neuropsychological Rehabilitation*.

[26] Farnè A, Rossetti Y, Toniolo S, Làdavas E (2002). Ameliorating neglect with prism adaptation. Visuo-manual and visuo-verbal measures. *Neuropsychologia*.

[27] Maravita A, McNeil J, Malhotra P, Greenwood R, Husain M, Driver J (2003). Prism adaptation can improve contralesional tactile perception in neglect. *Neurology*.

[28] Shiraishi H, Muraki T, Itou A, Hirayama K (2010). Prism intervention helped sustainability of effects and ADL performances in chronic hemispatial neglect: a follow-up study. *NeuroRehabilitation*.

[29] Cocchini G, Beschin N, Jehkonen M (2001). The fluff test: a simple task to assess body representation neglect. *Neuropsychological Rehabilitation*.

[30] McIntosh RD, Brodie EE, Beschin N, Robertson IH (2000). Improving the clinical diagnosis of personal neglect: a reformulated comb and razor test. *Cortex*.

[31] Wilson B, Cockburn J, Halligan PW (1987). *Behavioral Inattention Test*.

[32] Rode G, Rossetti Y, Boisson D (2001). Prism adaptation improves representational neglect. *Neuropsychologia*.

[33] McIntosh RD, Rossetti Y, Milner AD (2002). Prism adaptation improves chronic visual and haptic neglect: a single case study. *Cortex*.

[34] Vallar G, Burani C, Arduino L (2010). Neglect dyslexia: a review of the neuropsychological literature. *Experimental Brain Research*.

[35] Maguire AM, Ogden JA (2002). MRI brain scan analyses and neuropsychological profiles of nine patients with persisting unilateral neglect. *Neuropsychologia*.

[36] Fortis P, Maravita A, Gallucci M (2010). Rehabilitating patients with left spatial neglect by prism exposure during a visuomotor activity. *Neuropsychology*.

